# Effects of electrical stimulation‐induced resistance exercise training on white and brown adipose tissues and plasma meteorin‐like concentration in rats

**DOI:** 10.14814/phy2.14540

**Published:** 2020-08-18

**Authors:** Yuhei Amano, Yudai Nonaka, Reo Takeda, Yutaka Kano, Daisuke Hoshino

**Affiliations:** ^1^ Bioscience and Technology Program Department of Engineering Science The University of Electro‐Communications Chofu Japan; ^2^ Japan Society for the Promotion of Science (JSPS) Tokyo Japan

**Keywords:** beige adipocyte, brown adipocyte, mitochondria, muscle, uncoupling protein, white adipocyte

## Abstract

Chronic endurance exercise training induces morphological and metabolic alterations including mitochondrial biogenesis in white adipose tissue (WAT) and brown adipose tissue (BAT) in rodents. A myokine called meteorin‐like (Metrnl) is associated with morphological and metabolic adaptation and increased in blood after acute resistance exercise. However, the effects of chronic resistance exercise training (RT), which aims to increase muscle mass and strength, on WAT and BAT are unclear. Therefore, we aimed to clarify the effects of RT on morphological and metabolic parameters in WAT and BAT and on plasma Metrnl concentrations. We applied electrical stimulation to both legs of rats as RT three times a week for 4 weeks. RT reduced adipocyte size in subcutaneous WAT but induced no changes in mitochondrial and thermogenesis proteins. In BAT, peroxisome proliferator‐activated receptor gamma coactivator‐1 alpha (PGC‐1α) protein levels and mitochondrial content markers were significantly higher in the RT group compared with the control group. A significant positive correlation was found between the expression of PGC‐1α in BAT and plasma Metrnl concentrations. These results suggest that plasma Metrnl is associated with PGC‐1α and mitochondrial biogenesis in BAT. This study describes a potential role of RT in preventing metabolic diseases via altering WAT and BAT and increasing plasma Mertnl concentrations.

## Graphical abstract

We quantified the diameters of the minor axis of the adipocytes as an indicator of adipocyte size. The resistance training group had a higher percentage of smaller adipocytes whereas the control group had a higher percentage of larger adipocytes.

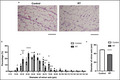

## 1 INTRODUCTION

Adipose tissue is an important organ for energy metabolism. There are three distinct types of adipocytes in the adipose tissues of rodents and humans: brown adipocytes in brown adipose tissue (BAT) and white and beige adipocytes in white adipose tissue (WAT). BAT is located primarily in the interscapular region (Cinti, 2005). BAT consists of multilocular brown adipocytes, which have numerous mitochondria and increased uncoupling protein 1 (UCP1) expression, which contributes to nonshivering thermogenesis (Cannon & Nedergaard, 2004). WAT is classified into subcutaneous (scWAT) and visceral WAT (vWAT) and is mainly composed of unilocular white adipocytes that have several physiological functions (Tran & Kahn, 2010). There are WAT depots throughout the body, but the location of WAT plays an important role in whole‐body metabolism. Beige adipocytes are found interspersed throughout the scWAT specifically. They have brown‐like phenotypes such as a multiculocator adipocyte and UCP1 expression (De Matteis et al., 2013; Wu et al., 2012) but these features are not at the same levels in beige adipose tissues as in classic brown adipose tissue. The different morphological and metabolic characteristics of each adipose tissue depot (BAT, vWAT, and scWAT) and each adipocyte (brown, white, and beige) contribute to systemic energy metabolism.

Chronic endurance exercise training is reported to induce morphological and metabolic alterations in the brown and white adipocytes of rodents. During the morphological alteration of white adipocytes, fat cell size is reduced after endurance training (Craig, Hammons, Garthwaite, Jarett, & Holloszy, 1981; De Matteis et al., 2013; Gollisch et al., 2009; Snook et al., 2017). With this reduction in cell size, the gene and protein expressions of UCP1 and mitochondrial biogenesis markers are increased in white adipocytes (Stallknecht, Vinten, Ploug, & Galbo, 1991; Stanford et al., 2015; Sutherland, Bomhof, Capozzi, Basaraba, & Wright, 2009; Trevellin et al., 2014). Importantly, along with increases in mitochondrial and thermogenic genes, metabolic improvements occur in the adipocytes (Craig et al., 1981; Snook et al., 2017; Stanford et al., 2015; Trevellin et al., 2014). Likewise, in brown adipocytes, endurance training increases the expression of UCP1 and peroxisome proliferator‐activated receptor gamma coactivator‐1 alpha (PGC‐1α), which is a master regular of mitochondrial biogenesis (De Matteis et al., 2013; Slocum et al., 2013; Xu et al., 2011). Therefore, endurance training causes changes in morphological and metabolic capacities, such as by increasing mitochondrial biogenesis in white and brown adipocytes. However, it is unclear whether resistance exercise training (RT), which aims to increase muscle mass and strength, influences morphology and the proteins involved with thermogenesis and mitochondrial biogenesis in these adipocytes.

The mechanism of endurance exercise training‐induced UCP1 and mitochondrial biogenesis is associated with increases in PGC‐1α (De Matteis et al., 2013; Sutherland et al., 2009; Xu et al., 2011). The increase in PGC‐1α expression is mediated by several myokines that are released from skeletal muscles during exercise (Boström et al., 2012; Carrière et al., 2014; Feldman, Streeper, Farese, & Yamamoto, 2006; Rao et al., 2014; Roberts et al., 2014). Among these molecules, there is a myokine called meteorin‐like (Metrnl) that increases the gene expression of UCP1 and is involved with mitochondrial biogenesis in white adipocytes (Rao et al., 2014). Plasma Metrnl concentrations increased in mice after acute downhill running exercise (Rao et al., 2014). Therefore, we hypothesized that chronic RT will increase Metrnl concentrations in the blood, induce morphological alterations in adipocytes, and stimulate the protein expressions of thermogenesis and mitochondrial markers in WAT and BAT. In this study, we aimed to clarify the effect of RT on morphological and metabolic parameters in WAT and BAT and to assess the blood levels of Metrnl using a rat RT model.

## 2 MATERIALS AND METHODS

### 2.1 Animals

Sixteen 10‐week‐old Wistar male rats were obtained from Japan SLC Inc. (Shizuoka, Japan). All animals were housed in duplicate in an environment maintained at 22°C–24°C with a 12:12‐hr light‐dark cycle and were allowed food and water ad libitum. Following a 3‐day acclimation period, the rats were randomly assigned to the control group (*n* = 8) or the RT group (*n* = 8). Body weight and amount of food intake were recorded three times a week. All experimental procedures were approved by the Institutional Animal Care and Use Committee of the University of Electro‐Communications (Tokyo, Japan; Approval No. 29).

### 2.2 Electrical stimulation for resistance training

Under isoflurane anesthesia, the hair was shaved off both the left and right lower leg of each rat in the RT group, and the shaved legs were cleaned with alcohol wipes. The rats were then positioned with both their left and right feet on a footplate (the ankle joint angle was positioned at 90°) in a prone position. The gastrocnemius muscles were stimulated percutaneously with electrodes (V‐120S3, Vitrode V, Nihon Kohden, Tokyo, Japan), which were cut into 10 × 5‐mm sections and connected to an electric stimulator and isolator (Model NS‐101, Unique Medical, Tokyo, Japan). The gastrocnemius muscles were isometrically exercised (3‐s stimulation × 10 contractions, with a 7‐s interval between contractions, for 5 sets with 3‐min rest intervals) under anesthesia. The voltage (~23 V) and stimulation frequency (100 Hz) were adjusted to produce maximal isometric tension. RT was performed three times a week for 4 weeks. This method has been confirmed to result in gastrocnemius muscle hypertrophy (Ogasawara et al., 2013, 2016). The control group was exposed to isoflurane anesthesia for five minutes three times a week for 4 weeks.

### 2.3 Tissue sample collection

Forty‐eight hours after the final bout of RT, the gastrocnemius muscles, scWAT in the inguinal region, epididymal WAT (eWAT), and BAT were carefully harvested from the rats in the control and RT groups under isoflurane anesthesia. We also collected blood from the heart and centrifuged it at 2,000 *g* for 20 min. Then, 2 ml of supernatant was collected for an enzyme‐linked immunosorbent assay (ELISA). All tissue and blood samples were frozen rapidly in liquid nitrogen and stored at −80°C.

### 2.4 Hematoxylin and eosin staining

The excised scWAT tissue blocks were frozen rapidly in isopentane cooled in liquid nitrogen and then stored at −80°C. With a cryostat (CM1950, Leica Biosystems, Wetzlar, Germany) at −30°C, four slices of 10‐µm sections were mounted on each polylysine‐coated glass slide (s‐9441, Matsunami, Osaka, Japan). Whole adipocyte sections were stained with H&E to examine the morphological changes. Images were taken and analyzed at ×10 (Plan Fluor 10×; Nikon, Tokyo, Japan). The diameter of the minor axis of the adipocytes was used as an indicator of adipocyte size to avoid the artifact created by the diagonal cutting. We analyzed 100–150 adipocytes per animal using ImageJ (U.S. National Institutes of Health, Bethesda, MD).

### 2.5 Western blot analysis

The adipose tissues were homogenized in cold RIPA lysis buffer (0.5 M Tris‐HCl, 1.5 M NaCl, 2.5% deoxycholic acid, 10% NP‐40, 10 mM EDTA, pH 7.4) supplemented with protease inhibitor cocktail using a beads crusher (uT‐01, Taitec, Saitama, Japan). Homogenized samples were centrifuged for 15 min at 10,000*g* at 4°C. After the homogenization, the infranatant was collected, and protein concentration was quantified using a bicinchoninic acid (BCA) protein assay kit (Thermo Fisher Scientific, Waltham, MA). Specific protein contents were determined by a Western blot analysis as described previously (Ikegami et al., 2019; Kitaoka et al., 2019). An equal amount of protein (10–40 µg) was loaded and separated on SDS‐PAGE gels. The proteins were transferred to nitrocellulose membranes by wet transfer (100 V, 75 min). The membranes were blocked for 1 hr in 3% skim milk dissolved in Tris‐buffered saline containing 0.1% Tween‐20 (TBS‐T). The membranes were incubated with the appropriate primary antibody (diluted in TBS‐T containing 3% skim milk) overnight at 4°C (anti‐OXPHOS (ab11413, Abcam, Cambridge, UK), anti‐PGC‐1α (AB3242, Merck Millipore, Burlington, MA), anti‐UCP1 (ab209483, Abcam), anti‐TH (#58844, CST Japan, Tokyo), and anti‐SERCA2 (#4388, CST Japan) antibodies, all diluted to 1:1,000). Following a 1‐hr incubation with the goat anti‐mouse or anti‐rabbit IgG‐linked secondary antibody, the bands were visualized using enhanced chemiluminescence reagent and quantified by densitometry (LAS‐3000, Fuji‐Film, Tokyo, Japan). Equal loading was confirmed by Ponceau S staining.

### 2.6 Quantitative real‐time PCR

Adipose tissues and gastrocnemius muscles were homogenized in cold TRIzol reagent (Thermo Fisher Scientific) using the beads crusher (uT‐01, Taitec). After the homogenization, total RNA was isolated using the RNeasy mini kit (Qiagen, Tokyo, Japan) and the RNA concentration was quantified using NanoDrop Lite (Thermo Fisher Scientific). Reverse transcription was conducted using the High‐Capacity RNA‐to‐cDNA Kit (Thermo Fisher Scientific) according to the manufacturer's instructions. Real‐time polymerase chain reaction (PCR) was performed with SYBR Green (Thermo Fisher Scientific) using the StepOne System (Thermo Fisher Scientific) in duplicate. 18S ribosomal RNA was used as an internal control. The fold changes were calculated on the basis of the ΔΔCt method. The following primers were purchased from Takara (Tokyo, Japan) and used: 18S ribosomal RNA, forward, 5′‐AAGTTTCAGCACATCCTGCGAGTA‐3′, and reverse, 5′‐TTGGTGAGGTCAATGTCTGCTTTC‐3′; UCP1, forward, 5′‐TGTGCAATGACCATGTACACCAA‐3′, and reverse, 5′‐GCACACAAACATGATGACGTTCC‐3′; Metrnl, forward, 5′‐CTTGCCATCTGCACCAGTGA‐3′, reverse, 5′‐TGCTGTTCTGGTACATGGGTGA‐3′.

### 2.7 ELISA

Circulating Metrnl concentrations were quantified using an ELISA kit (OKEH00577, Aviva Systems Biology, San Diego, CA), according to the manufacturer's instructions. Optical density was measured at 450 nm using a microplate reader (Thermo Fisher Scientific).

### 2.8 Statistical analysis

All data were presented as the mean ± standard error of the mean. A two‐way repeated measures analysis of variance (ANOVA) was used to analyze the differences in body weight (days and training). A two‐way ANOVA was used to analyze the differences in adipocyte size (training and range of diameters). Post hoc comparisons were performed using the Sidak procedure. Other data were analyzed using a two‐tailed unpaired Student's *t*‐test. All statistical analyses were performed using GraphPad Prism version 8.4 Software (GraphPad, San Diego, CA, USA). *p* < .05 was considered significant.

## 3 RESULTS

### 3.1 Body and tissue weights

**FIGURE 1:**
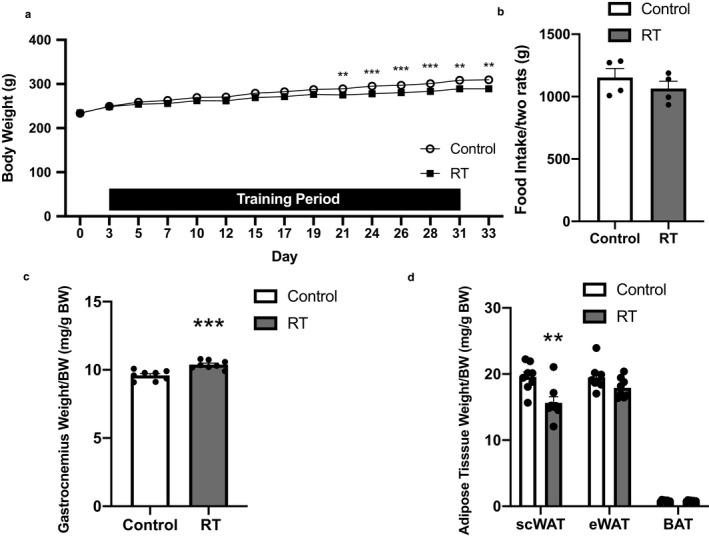
The effects of 4 weeks of resistance training on body weight (a), total food intake (b), muscle weight (c and d), and adipose tissue weights (e and f) in rats. RT, resistance training group; BW, body weight; scWAT, inguinal subcutaneous white adipose tissue; eWAT, epididymal white adipose tissue; BAT, brown adipose tissue. *n* = 8 in each group except for B (*n* = 4). Mean ± *SEM*. *p < .05, **p < .01, ***p < .001

Body weight was significantly decreased in the RT group after 4 weeks of RT (*p* < .05, Figure 1a); conversely, total food intake did not differ significantly between the two groups (Figure 1b). Although the absolute gastrocnemius muscle weight did not differ significantly (control: 2.967 ± 0.032 g, RT: 3.002 ± 0.0340 g), the relative weight of the gastrocnemius muscle according to body weight in the RT group was significantly higher than that in the control group (*p* < .001, Figure 1c). The absolute weights of the scWAT and eWAT in the RT group were significantly lower than those in the control group (*p* < .05, scWAT, control: 5.994 ± 0.228 g, RT: 4.794 ± 0.279 g; eWAT: control: 6.039 ± 0.244 g, RT: 5.180 ± 0.157 g), but the weight of the BAT did not differ (control: 0.248 ± 0.014 g, RT: 0.230 ± 0.009 g). The relative weight of the scWAT, but not of the eWAT or BAT, was significantly lower in the RT group than in the control group (*p* < .01, Figure 1d).

### 3.2 Adipocyte size

**FIGURE 2:**
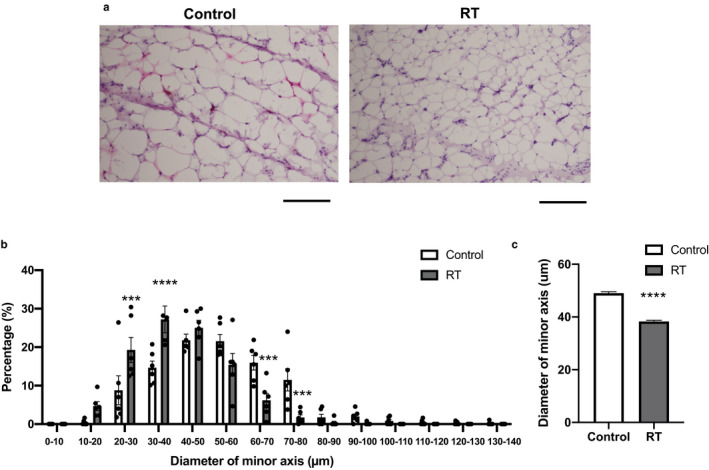
The effects of 4 weeks of resistance training on inguinal subcutaneous white adipocytes. The representative images of H&E stained inguinal subcutaneous white adipocytes of the control (left panel) and trained (right panel) rats (a). Histogram of the diameter of the minor axis of the adipocytes (b). Average value of the diameter of the minor axis (c). A total of 1,020 and 1,108 adipocytes were analyzed in the control and the RT groups, respectively. The black scale bar indicates 200 µm. RT; resistance training group. *n* = 8 animals in each group. Mean ± *SEM*. ***p < .001, ****p < .0001

Because the relative weights of scWAT in the RT group were significantly decreased, we examined the adipocyte size of scWAT using hematoxylin and eosin (H&E) staining. According to the H&E staining images, the inguinal subcutaneous white adipocytes appeared smaller in the RT group after 4 weeks of RT (Figure 2a). Then, we quantified the diameters of the minor axis of the adipocytes as an indicator of adipocyte size. The RT group had a higher percentage of smaller adipocytes whereas the control group had a higher percentage of larger adipocytes (*p* < .001, Figure 2b). The average adipocyte size in the RT group was significantly smaller than that of the control group (*p* < .001, Figure 2c).

### 3.3 Protein expression levels involved with mitochondria and thermogenesis in scWAT, eWAT, and BAT

**FIGURE 3:**
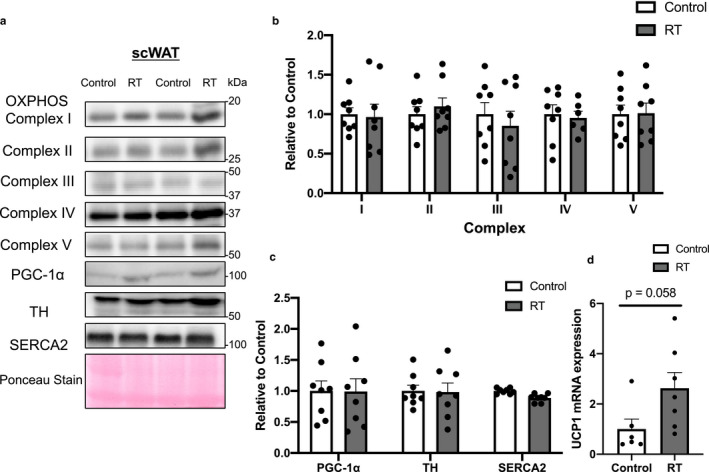
The effects of 4 weeks of resistance training on protein expression in inguinal subcutaneous white adipose tissue (scWAT). Representative Western blot images of each specific band (a). Protein contents of OXPHOS (b), PGC‐1α, TH, and SERCA2 (c) in scWAT. Ponceau staining was used as a loading control. UCP1 mRNA expression in scWAT (D). *n* = 6–8 in each group. Mean ± *SEM*

**FIGURE 4:**
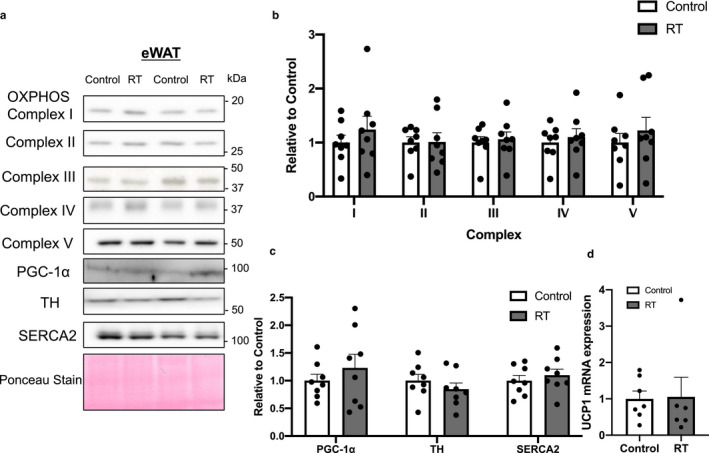
The effects of 4 weeks of resistance training on protein expression in epididymal white adipose tissue (eWAT). Representative Western blot images of each specific band (a). Protein contents of OXPHOS (b), PGC‐1α, TH, and SERCA2 (c) in eWAT. Ponceau staining was used as a loading control. UCP1 mRNA expression in eWAT (D). *n* = 6–8 in each group. Mean ± *SEM*

**FIGURE 5:**
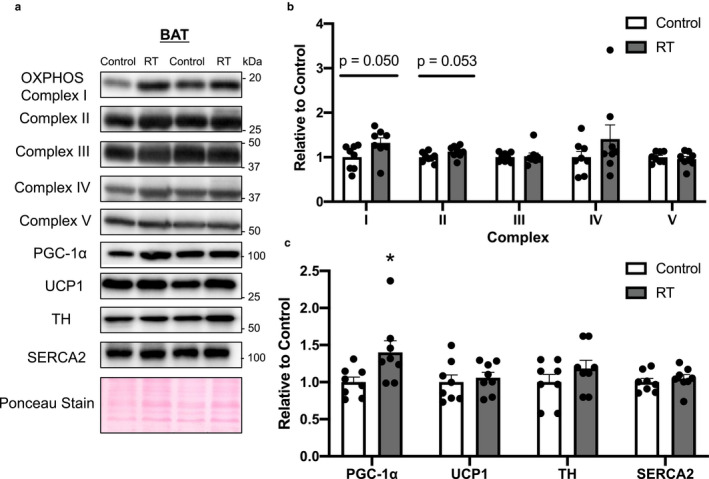
The effects of 4 weeks of resistance training on protein expression in brown adipose tissue (BAT). Representative Western blot images of each specific band (a). Protein contents of OXPHOS (b), PGC‐1α, UCP‐1, TH, and SERCA2 (c) in BAT. Ponceau staining was used as a loading control. *n* = 8 in each group. Mean ± *SEM*. *p < .05

The OXPHOS, PGC‐1α, tyrosine hydroxylase (TH), and sarco(endo)plasmic reticulum Ca^2+^‐ATPase (SERCA)2 protein levels in scWAT and eWAT did not differ significantly between the two groups (scWAT in Figure 3a–c, eWAT in Figure 4a–c). In BAT, the protein levels of complexes I and II, but not III, IV, or V, of OXPHOS, which are mitochondrial enzymes, tended to increase after 4 weeks of RT (complex I, *p* = .050; complex II, *p* = .053, Figure 5b). Furthermore, the protein levels of PGC‐1α, a master regulator of mitochondrial biogenesis, were significantly higher in the RT group than in the control group (*p* < .05, Figure 5c) whereas the UCP1, TH, and SERCA2 protein levels did not differ significantly between the two groups.

### 3.4 UCP1 mRNA expression

UCP1 mRNA expression in scWAT, not in eWAT, tended to be higher in the RT than in the control group (*p* = .058, scWAT in Figure 3d, eWAT in Figure 4d).

### 3.5 Plasma Metrnl concentration and muscle Metrnl mRNA expression

**FIGURE 6:**
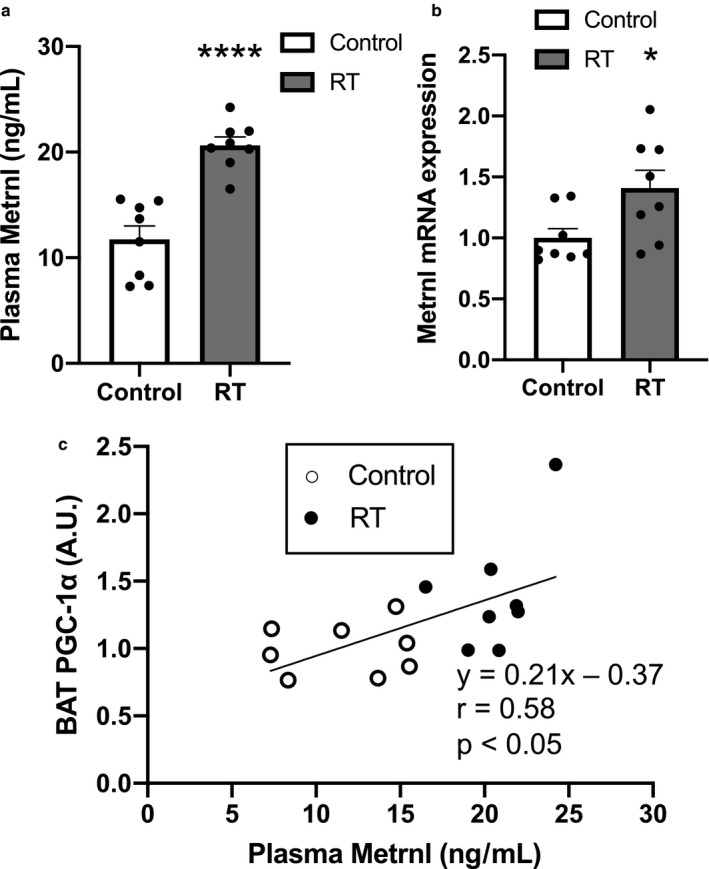
The effects of 4 weeks of resistance training on plasma Metrnl levels (a) Metrnl mRNA expression in gastrocnemius muscle (b). The correlation between plasma Metrnl levels and PGC‐1α protein contents in BAT (c). *n* = 8 in each group. Mean ± *SEM*. *p < .05, ****p < .0001

The plasma Metrnl concentration was significantly higher in the RT group than in the control group (*p* < .0001, Figure 6a). The gastrocnemius muscle Metrnl mRNA expression was significantly higher in the RT group than in the control group (*p* < .05, Figure 6b). The positive correlation between plasma Metrnl concentrations and PGC‐1α protein levels in BAT is displayed (Figure 5c, *p* < .05, *r* = .58).

## 4 DISCUSSION

We examined the effects of RT on adipocyte size in scWAT and levels of proteins involved with mitochondria and thermogenesis in WAT and BAT. We performed electrical stimulation‐induced RT on rats 3 days/week for 4 weeks. We found that RT reduced the adipocyte size of scWAT but did not alter the mitochondrial content markers and UCP1 gene expression. Interestingly, RT increased PGC‐1α and mitochondrial content markers in BAT, concomitantly with an increase in plasma Metrnl concentrations. These results suggest that plasma Metrnl concentrations may be associated with PGC‐1α protein expression and mitochondrial biogenesis in BAT.

We had hypothesized that 4 weeks of RT would result in morphological and metabolic alterations in rat white adipocytes. Because RT decreased the weight of scWAT but not eWAT, relative to body weight, we analyzed the adipocyte size of scWAT. The data showed that RT reduced adipocyte size, as evaluated by the diameters of the minor axis of each adipocyte in scWAT. This is consistent with the previous study that the same period of endurance training with wheel running exercise also reduced fat cell size in scWAT in rats (Gollisch et al., 2009). However, exercise mode is totally different because we used local muscle contraction‐based resistance exercise, which we would expect to result in relatively lower energy expenditure than endurance exercise. We also observed no significant difference in total food intake between the two groups in this study. We speculate that the reduction in adipocyte of scWAT was associated with an increase in plasma Metrnl concentration. It is reported that increased plasma Metrnl concentration changed adipocyte directly, such as increases in thermogenic and mitochondrial gene expression in scWAT (Rao et al., 2014). Others also found that Metrnl directly upregulated lipolysis and lipogenesis gene expression in 3T3‐L1 adipocytes (Li et al., 2015). Therefore, local muscle contraction‐induced secreted Metrnl possibly activates turnover of lipid in adipocytes of scWAT. This possibility would be associated with inguinal fat weight loss locally. However, Metrnl is not only the factor to induce adaptation of fat tissues involved with lipid metabolism after exercise training. It is well known that there are other myokines and metabolites secreted from working muscles during exercise, including musclin (Subbotina et al., 2015), irisin (Boström et al., 2012), myostatin (Feldman et al., 2006), lactate (Carrière et al., 2014), and β‐Aminoisobutyric acid (Roberts et al., 2014).

Unlike the morphological alteration of adipocytes in scWAT, mitochondrial marker proteins and UCP1 gene expression did not significantly change in the scWAT. PGC‐1α and TH, which is the limiting enzyme for catecholamine production (Flatmark, 2000), and SERCA2, which is another thermogenesis regulator (Ikeda et al., 2017), were also not altered. Only one previous study has examined the effects of RT on UCP1 mRNA expression in rat scWAT (Reisi, Ghaedi, Rajabi, & Marandi, 2016). They found that 8 weeks of ladder climbing increased UCP1 mRNA expression levels, but they did not examine mitochondrial markers and PGC‐1α levels. With endurance training, Wu et al. found that 8 weeks of treadmill running increased PGC‐1α and UCP1 mRNA expression levels and palmitate oxidation in rat scWAT (Wu, Bikopoulos, Hung, & Ceddia, 2014). Trevellin et al. also reported that 30 days of swimming exercise increased the protein levels of PGC‐1α and cytochrome oxidase IV (COXIV), a mitochondrial marker, in mouse scWAT (Trevellin et al., 2014). The resistance training exercise used in this study was different than the exercises described in the studies above, with local muscle contraction for 4 weeks rather than whole‐body endurance training. According to earlier studies, this RT program would be enough to increase muscle mass (Ogasawara et al., 2013, 2016). Therefore, a longer period of training and/or more whole‐body exercise might stimulate increases in the protein and gene expression involved with mitochondrial biogenesis and thermogenesis in scWAT.

Interestingly, RT increased PGC‐1α and mitochondrial content markers in the BAT, suggesting that there are increases in mitochondrial biogenesis and oxidative capacity in the BAT after RT. Endurance training has been shown to increase mitochondrial biogenesis markers and UCP1 mRNA levels in the BAT of mice (Slocum et al., 2013; Xu et al., 2011) and rats (De Matteis et al., 2013). PGC‐1α is a master regulator of mitochondrial biogenesis that induces transcriptional activation of mitochondrial genes with key transcriptional factors such as NRF1,2 and MEF2 (Scarpulla, 2011). Indeed, Xu et al. reported that treadmill training increased mitochondrial numbers in BAT evaluated by transmission electron microscopy images (Xu et al., 2011). Although we did not measure mitochondrial volume or contents directly, we estimate that mitochondrial contents would increase in BAT because several OXPHOS proteins increased in our study. Despite increases in OXPHOS protein levels, UCP1 protein levels did not change after 4 weeks of RT. This may be a contradictory result with increased mitochondrial contents because UCP1 proteins localize within mitochondria. Several previous studies have demonstrated that the UCP1 mRNA expression levels of BAT were remarkably reduced in rodents exposed to chronic endurance training (Wu et al., 2014; Yamashita et al., 1993). Therefore, changes in the transcription levels of UCP1 have not been consistent among previous studies. Moreover, there have been a report of changes in UCP1 protein levels without changes in COXIV protein, a mitochondrial content marker (Okamatsu‐Ogura et al., 2017). Taken together, it is unclear why there were different adaptations of mitochondrial markers and UCP1 protein levels in this study, but the 4‐week RT increased mitochondrial markers without changing UCP1 protein levels in BAT in rats.

We assessed the plasma Metrnl concentration to elucidate the mechanisms of increases in PGC‐1α and mitochondrial marker proteins in BAT after RT because it has been reported that Metrnl released from skeletal muscles increases PGC‐1α and mitochondrial gene expression in WAT (Rao et al., 2014). As a result, the chronic 4‐week RT significantly increased plasma Mertnl concentrations. This is consistent with a previous study showing that chronic endurance training increased plasma Mternl concentrations in normal diet‐fed mice (Bae, 2018). We estimate that the increase in Metrnl in the blood is due to the increase in Metrnl in skeletal muscles. This is supported by our data showing that muscle Metrnl mRNA expression showed a slight but significant increase after 4 weeks of RT and previous studies showing that 4‐week endurance exercise training increased muscle Metrnl protein contents in mice (Bae, 2018). Furthermore, in a human study, acute and chronic high‐intensity interval exercise increased muscle Metrnl mRNA expression levels (Eaton et al., 2018). In our study, there was a significant positive correlation between PGC‐1α protein levels in BAT and plasma Metrnl concentrations. These results suggest that increased plasma Metrnl released from the skeletal muscle is associated with PGC‐1α and mitochondrial biogenesis in BAT. However, the causal relationship between Metrnl and PGC‐1α in the BAT was not clarified in this study. Metrnl stimulated PGC‐1α expression via increases in catecholamine secretion and TH expression (Rao et al., 2014). Because TH did not change after RT in this study, another mechanism of PGC‐1α expression by Metrnl would exist.

RT is a typical training that aims to increase muscle mass and strength. Additionally, this study provides evidence that RT could prevent metabolic diseases via alterations in WAT and BAT with concomitant increases in the plasma Mertnl concentration. WAT expansion is critical for the development of obesity via the enlargement of adipocytes (Parlee, Lentz, Mori, & MacDougald, 2014). Smaller white adipocytes have a higher glucose uptake, oxidation capacity (Craig et al., 1981) and lipolysis (Snook et al., 2017), which all improve metabolic function. Maintenance of small white adipocytes can help prevent obesity. Mitochondrial adaptation in BAT also improves whole‐body metabolism, such as lipid utilization (Slocum et al., 2013). Changes in adipose tissue itself due to chronic endurance training improve muscle glucose uptake and impairments in systemic glucose metabolism (Stanford et al., 2015). On the other hand, although scWAT is highly responsive to exercise, its role is not necessary for the endurance training‐induced improvement of whole‐body glucose metabolism in mice (Peppler, Townsend, Knuth, Foster, & Wright, 2018). More recent work has demonstrated that Metrnl directly improves glucose tolerance in skeletal muscle cells (Lee et al., 2020). Future studies will need to elucidate whether the RT‐induced alteration of brown and white adipocytes ameliorates metabolic dysfunction in obesity and metabolic diseases.

This study has a potential limitation: Recent work (McKie et al., 2019; Raun et al., 2020) has demonstrated that housing mice at subthermal neutral conditions, that is, room temperature, rather than at thermal neutrality can stimulate increases in mitochondrial and thermogenic proteins in adipose tissues with endurance exercise training. Housing temperature (22–24°C) may have impacted our data because the thermoneutral zone of Wistar rats is in the range of 29.5–30.5°C (Romanovsky, Ivanov, & Shimansky, 2002). There are no studies have examining how housing temperature can impact the effects of RT on adipose tissues, so this issue should be elucidated in future studies.

In conclusion, this study revealed that RT by electrical stimulation three times a week for 4 weeks increased muscle weight relative to body weight and decreased the weight of scWAT. The RT reduced the adipocyte size of scWAT but did not alter mitochondrial and thermogenesis protein levels. PGC‐1α and mitochondrial marker proteins were increased in BAT, along with the increased plasma concentration of Metrnl. This study suggests a potential role of RT to prevent metabolic diseases via alterations of white and brown adipose tissues including increases in plasma Mertnl concentrations.

## CONFLICT OF INTEREST

The authors declare no conflict of interest.

## AUTHOR CONTRIBUTIONS

YA and DH were involved in conception and design of research, prepared figures, and drafted the manuscript. YA, YN, RT, and DH performed experiments. YA, YN, RT, YK, and DH analyzed and interpreted the data, edited and revised the manuscript, and approved the final version of manuscript.
